# Association between reproductive history and menopausal timing: exploring rural-urban differences in a cross-sectional survey

**DOI:** 10.1186/s12905-026-04441-y

**Published:** 2026-04-06

**Authors:** Ossai Onyinyechi Gift, Madu Ignatius Ani

**Affiliations:** Department of Geography and Environmental Sustainability, Faculty of the Social Sciences, University of Nigeria, Nsukka, Enugu State Nigeria

**Keywords:** Menopause, Menopausal timing, Rural-urban differences, Reproduction, Reproductive history, Women

## Abstract

**Background:**

As women spend nearly one-third of their lives postmenopausal, this transition warrants greater public health attention. Menopausal timing is affected by biological, lifestyle, and socioeconomic characteristics of women. While previous studies emphasize biomedical correlations, few account for how urban-rural differences and reproductive health variables influence menopausal timing. This study examines the influence of reproductive history on menopausal timing among women in urban and rural areas in Nigeria and provides evidence that can help target interventions aimed at improving midlife and post-reproductive health.

**Methods:**

Data were collected from 294 women (181 urban, 113 rural) using structured questionnaires from March to June 2025. Descriptive, chi-square, and multinomial logistic regression analyses were used to describe, determine associations, and predictors of menopausal timing (early, normal, late) based on reproductive history. Data analysis was carried out in SPSS. Menopausal timing was the dependent variable, and independent variables included reproductive history with multiple variables such as menarche, number of pregnancies, number of children, breastfeeding, family planning, and miscarriages, while controlling for the area type (i.e., urban or rural).

**Results:**

Among the 294 respondents, mean age at menopause was 49.6 years and modal age was 48 years. Overall, 5.5% of women experienced early menopause (42–44 years), 77.2% experienced normal menopause (45–53 years), and 17.3% experienced late menopause (54–60 years). Stratified by residence, 7.2% of urban women and 2.7% of rural women had early menopause, while 20.0% of urban and 13.3% of rural women experienced late menopause. Chi-square analyses indicated significant associations (*p* < 0.05) between menopausal timing and reproductive history variables such as age at menarche, number of pregnancies and children, breastfeeding history, use of family planning, and history of miscarriage among urban women. In rural women, similar associations were observed, except for menstrual regularity, breastfeeding status, and duration of breastfeeding, which were not statistically significant. Regression analysis showed that in urban areas, early menarche (OR = 8.75, *p* < 0.05) and a higher number of children (OR = 49.3, *p* < 0.05) significantly increased the odds of early menopause, whereas a higher number of pregnancies reduced the likelihood of late menopause (OR = 0.69, *p* < 0.05). In rural areas, only the number of pregnancies was significantly associated with late menopause (OR = 1.85, *p* < 0.05). Reproductive history exerted greater influence in urban settings than in rural environments.

**Conclusion:**

Menopausal timing among women in the study area occurred predominantly within the normal age range, although variations were observed between urban and rural populations. The findings indicate that reproductive history factors, particularly age at menarche, number of pregnancies, and number of children, play an important role in menopausal timing, with stronger associations observed among urban women. These results highlight the importance of incorporating reproductive life-course factors into women’s health policies and interventions aimed at improving midlife reproductive health and menopause related care in Nigeria.

**Supplementary Information:**

The online version contains supplementary material available at 10.1186/s12905-026-04441-y.

## Background

Menopause has far-reaching implications for women’s health. Early menopause is associated with elevated risks of cardiovascular disease, stroke, and osteoporosis, while late menopause increases the likelihood of hormone-related cancers such as breast and endometrial cancer [1]. Given that women now spend nearly one-third of their lives postmenopausal [2–5], the health burden and quality of life associated with menopausal transition deserve greater public health attention [6, 7].

Menopause represents a significant physiological milestone in a woman’s life, signaling the end of ovarian follicular function and permanent cessation of menstruation and fertility [8]. Clinically, it is confirmed retrospectively following twelve consecutive months without menstruation, provided the amenorrhea is not due to surgical intervention or underlying pathology [8, 9]. Approximately 25 million women transition through menopause each year, with the global population of postmenopausal women projected to reach 1.2 billion by 2030. Over 75% of women experience menopausal symptoms, and a significant portion, around 25%, describe them as severe [7]. Although menopause is a universal biological transition, the age at which it occurs varies across populations, influenced by an interplay of genetic, environmental, and lifestyle factors [4, 10].

In developed countries, the average age of menopause is approximately 51 years, while in developing countries, it ranges between 43 and 49 years, as reported in various regional studies [8]. In Nigeria, considerable regional variability exists; mean ages at menopause include 48.4 ± 5.2 years in Enugu [11], 46.71 ± 4.57 years in Ado-Ekiti [12], and 44.23 ± 2.74 years in Zuturung district [13]. These disparities suggest that both biological and contextual factors significantly shape menopausal timing.

The onset of menopause is governed by declining estrogen and progesterone levels due to ovarian senescence, accompanied by shifts in gonadotropins such as follicle-stimulating hormone (FSH) and luteinizing hormone (LH) [14]. While the genetic component is central, a significant proportion of variance in age at natural menopause is attributable to non-genetic influences, including socioeconomic status (SES), reproductive history, and lifestyle behaviors [15, 16]. Recent studies have claimed that socioeconomic factors such as education, occupation, income, and access to healthcare play a crucial role in shaping the menopausal experience and the age at natural menopause.

Existing literature consistently associates lower socioeconomic status (SES) with earlier onset of menopause and increased symptom severity [17–19]. For instance, women with lower education and income levels, or those engaged in manual labor or unemployed, report more intense menopausal symptoms, including depression, fatigue, and musculoskeletal discomfort [20]. Furthermore, women who are less informed about hormone replacement therapy (HRT) or alternative therapies are often less equipped to manage these symptoms, particularly those in rural or socioeconomically disadvantaged settings [21]. Reproductive history variables such as age at menarche, parity, and contraceptive use are especially salient as proxies for cumulative estrogen exposure and thus affect both the timing of menopause and the experience of symptoms [16, 22].

Among these factors, reproductive history encompassing age at menarche, parity, and contraceptive use plays a crucial role in determining menopausal age. These reproductive markers serve as indicators of lifetime estrogen exposure and are strongly correlated with the timing and symptoms of menopause [14, 16]. Socioeconomic characteristics such as income, education, occupation, and living conditions further modify this trajectory, with women of lower socioeconomic status generally experiencing menopause earlier and more severely [15, 17]. Furthermore, lifestyle factors such as smoking, alcohol consumption, and physical activity also exert independent effects on menopausal timing [19].

Importantly, menopausal transition is not experienced uniformly across different geographies. Urban and rural environments differ in healthcare access, environmental exposures, occupational stressors, and cultural norms, factors that may contribute to variation in the age and experience of menopause [23]. While urban areas often offer improved health infrastructure and education, they may also expose women to pollutants and stressors that could hasten reproductive aging. Conversely, rural environments might promote natural lifestyles but often lack adequate reproductive health services. These contextual differences remain underexplored in literature, particularly in Sub-Saharan Africa.

Although several studies have examined reproductive factors associated with menopause [8, 17, 24, 25], few have investigated how these relationships manifest across urban and rural populations. In Nigeria, where sociocultural beliefs and limited health education shape reproductive behaviors and perceptions, understanding these rural-urban disparities is especially critical. Most existing research focuses either on urban areas or lacks the depth needed to inform policy at the community level [1, 26]. Furthermore, studies rarely consider how infertility, parity beyond two children, or socioeconomic inequities intersect to influence menopausal outcomes in these distinct settings.

This study addresses these gaps by investigating the association between reproductive history and the timing of menopause, with particular attention to differences between rural and urban populations. Through a cross-sectional survey of menopausal women, the research aims to generate population-specific insights that can guide culturally sensitive, geographically responsive public health interventions in Nigeria and similar settings. To achieve its aim, the following objectives were pursued: examine the demographic and reproductive characteristics of respondents and determine the average age at menopause, identify specific reproductive factors that are associated with menopausal timing, and assess the influence of reproductive history on menopausal timing and type.

## Methods

This study employed a community-based cross-sectional design to examine the relationship between reproductive history and menopausal timing among women in Enugu State, Nigeria. A mix of urban, semi-urban and rural populations, diverse reproductive histories, and varying levels of access to healthcare services characterizes the state. This will provide a vast data on understanding how reproductive history influence the timing of menopause among rural and urban women in Nigeria.

The sampling process was guided by a statistically determined sample size using the formula proposed by Taro Yamane. The formula $\:n=N/[1+N(e{)}^{2}]$ was used, where $\:n$ represents the sample size, $\:N$ denotes the finite population, and $\:e$ represents the level of precision or sampling error [27]. For this study, a 95% confidence level with a precision level of 0.05 was adopted. Due to the absence of reliable demographic data specifically on menopausal women, the total female population of Enugu State (*N* = 2,507,512) was used as a proxy population for the computation. Applying the formula yielded a target sample size of 400 respondents. To reflect Nigeria’s demographic distribution, where approximately 53.52% of the population resides in urban areas [28], the study initially aimed to survey 214 urban women and 186 rural women who had reached menopause.

The selection of the study areas was structured to capture both rural and urban areas within the state. Two major urban environments, Enugu Urban and Nsukka Urban, were selected to represent urban populations due to their relatively higher concentration of health facilities and population density. In contrast, rural representation was obtained from communities including Eha-Amufu and other rural settlements located within three local government areas of the state. This spatial selection allowed the study to compare menopausal experiences and healthcare access between women living in different settlements.

Data was collected over three months, from March to May 2025, across both rural and urban communities. The study population consisted of postmenopausal women aged 42 to 69 years who had experienced natural menopause and were selected purposively. Although the initial target was to administer 400 questionnaires, only a total of 294 participants (181 urban women and 113 rural women) who were purposively selected from households and community clusters, based on their availability and willingness to participate, were included. Inclusion criteria required that participants had experienced spontaneous amenorrhea for at least 12 consecutive months, in accordance with the World Health Organization’s (WHO) definition of natural menopause. Women were excluded if they had undergone medically or surgically induced menopause, including hysterectomy, oophorectomy, chemotherapy, or radiotherapy.

Data was collected through face-to-face administration of a pretested structured questionnaire. Survey were conducted by trained research assistants in private settings to ensure comfort and confidentiality. The questionnaire was designed in two sections. The first section captured socio-demographic data; menopausal age, including age, marital status, educational level, occupation, monthly income, and residential location (urban or rural). The second section gathered information on reproductive history. The study obtained variables from previous studies [16, 25, 29], especially from the variables include age at menarche, age at first pregnancy and childbirth, parity (number of pregnancies and live births), history of abortions (spontaneous or induced), and use of hormonal contraceptives. Participants were asked to report the age at which this occurred, and their menopausal status was confirmed through the questionnaire responses.

The timing of menopause was categorized as early, normal, or late, as also adopted by [29] but for normal categorization. Menopausal timing was calculated based on the mean age at menopause using the mean ± standard deviation as a guideline, ages falling within this interval (mean) were considered to represent the typical menopausal age among the respondents. Consequently, menopause occurring before age (-SD of the mean age ) was categorized as early menopause, while menopause occurring after age (+ SD of the mean age) was classified as late menopause. This classification allowed the study to identify variations in menopausal timing relative to the central tendency of the sample. The use of the mean ± standard deviation is a commonly applied statistical approach for identifying the normal range of a variable within a population distribution [29].

The questionnaire was developed specifically for this study. An English language version was uploaded as a supplementary file. Ethical approval for this study was granted by the Strategic Contacts, Ethics, and Publication (STRACEP) Office at the University of Nigeria, Nsukka, Enugu state Nigeria (Reference number: UNN/EC/NSTA/FSS-GGY-RG/011/31-JULY/2025), and in compliance with the Helsinki Declaration. Informed consent was obtained from all participants before data collection. Respondents were fully briefed on the objectives of the study, the voluntary nature of participation, and their right to withdraw at any stage without penalty. To protect respondent’s privacy, anonymity, and confidentiality were strictly maintained throughout the research process, and data were used solely for academic purposes.

Data analysis was conducted using the Statistical Package for the Social Sciences (SPSS) version 25.0. Descriptive statistics, including means, standard deviations, frequencies, and percentages, were used to summarize the socio-demographic and reproductive characteristics of participants. Chi-square tests were employed to examine associations between categorical variables. Multinomial logistic regression analysis was used to explore the relationships between reproductive history indicators and the timing of menopause. Statistical significance was set at *p* < 0.05. Results were presented using tables, graphs, and narrative descriptions.

## Results

### Demographic and reproductive history characteristics of respondents

The study involved 294 menopausal women aged 45 to 69 years, with a greater proportion residing in urban areas (61.6%) compared to rural areas (38.4%). In Table 1, the average age at menopause across the sample was 49.6 years, with urban women reporting a slightly lower mean age of 49.3 years, while their rural counterparts reported a higher mean of 49.9 years. The most frequently reported menopausal age range was 47–48 years, particularly among urban women (35.3%). Rural women, however, had a more even distribution, with higher proportions reaching menopause at later ages; 53–54 years (10.6%) and 59–60 years (6.2%), suggesting delayed menopausal onse in some rural populations.

**Table 1:** Demographic and reproductive history characteristics of menopausal women in urban and rural areas

Variables	Total level F(%) *N* = 294	Urban F(%) *N* = 181 (61.6)	Rural F(%) *N* = 113 (38.4)
Age at Menopause (Mean)	49.6±4.0	49.3±3.9	49.9±4.3
* 42–43*	16(5.5)	13(7.2)	3(2.7)
* 45–46*	42(14.3)	20(11.1)	22(19.5)
* 47–48*	85(28.9)	64(35.3)	21(18.5)
* 49–50*	47(16.0)	25(13.8)	22(19.5)
* 51–52*	40(13.6)	22(12.1)	18(16.0)
* 53–54*	14(4.7)	2(1.2)	12(10.6)
* 55–57*	43(14.6)	35(19.4)	8(7.1)
* 59–60*	7(2.4)	0(0.0)	7(6.2)
Current Age (mean)	57.3	56.5	58.4
* 45–49*	14(4.8)	7(3.9)	7(6.2)
* 50–54*	98(33.3)	72(39.8)	26(23.1)
* 55–59*	77(26.3)	47(26.1)	30(26.6)
* 60–64*	56(19.10	26(14.4)	30(26.6)
* 65–69*	49(16.7)	29(16.1)	20(17.7)
Marital Status			
* Married*	266(90.5)	154(81.5)	112(99.1)
* Single*	14(4.8)	14(7.7)	
* Divorced*	14(4.8)	13(7.2)	1(0.9)
Highest Educational Qualification			
* None*	21(7.1)	3(1.7)	18(15.9)
* Primary*	42(14.3)	17(9.4)	25(22.1)
* Secondary*	49(16.7)	33(18.2)	16(14.2)
* Tertiary*	182(61.9)	128(70.7)	54(47.8)
Age at Menarche			
* 11–12*	70(26.3)	60(33.9)	10(11.2)
* 13–14*	91(34.3)	63(35.6)	28(31.5)
15–16	91(34.3)	42(23.7)	49(55.0)
* 17–18*	7(2.6)	5(2.8)	2(2.2)
* 19–20*	7(2.6)	7(4.0)	0(0.0)
Regularity of the Menstrual cycle			
* Very regular*	25.9(88.1)	153(84.5)	106(93.8)
* Irregular*	35(11.9)	28(15.5)	7(6.2)
Number of pregnancies			
* 0*	14(4.9)	14(8.0)	0(0.0)
* 1–2*	7(2.4)	1(0.6)	6(5.4)
* 3–4*	56(19.5)	41(23.4)	15(13.4)
5–6	105(36.6)	52(29.7)	53(47.3)
* 7–8*	91(31.7)	59(33.7)	32(28.6)
* 9–10*	14(4.8)	8(4.6)	6(5.4)
Number of Children			
* 0*	14(4.8)	14(7.7)	0(0.0)
* 1–2*	28(9.5)	20(11.1)	8(7.1)
3–4	84(28.6)	61(33.7)	23(20.3)
* 5–6*	154(52.4)	85(46.9)	69(61.0)
* 7–8*	14(4.8)	1(0.6)	13(11.5)
Breastfeeding			
* No*	14(4.76)	13(7.5)	1(0.9)
* Yes*,* all my children*	273(95.1)	161(92.5)	112(99.1)
Period of Breastfeeding			
* None*	14(4.76)	13(7.18)	1(0.9)
* 6 months or less*	7(2.4)	6(3.3)	1(0.9)
* 6–11 months*	28(9.5)	25(13.8)	3(2.7)
* One year and above*	238(81.0)	130(71.8)	108(95.6)
Family Planning			
* No*	224(76.2)	137(75.7)	87(77.0)
* Yes*	70(23.8)	44(24.3)	26(23.0)
Methods of Family planning used			
* Natural*	14(18.2)	12(24.0)	2(7.4)
* Hormonal Method*	28(36.4)	25(50.0)	3(11.1)
* Barrier Method*	7(9.1)	0(0.0)	7(25.9)
* Non-Hormonal Method*	28(36.4)	13(26.0)	15(55.6)
History of Miscarriage			
* No*	189(65.9)	107(61.1)	82(73.2)
* Yes*	98(34.1)	68(38.9)	30(26.8)

The average age of respondents was 57.3 years. Rural women tended to be older, with greater representation in the 60–64 (26.6%) and 65–69 (17.7%) age brackets, compared to urban women whose largest age group was 50–54 years (39.8%). Marital status varied notably across residence; while the majority of women in both settings were married, widowhood was more prevalent among rural women (33.6%) than among those in urban areas (13.8%), possibly indicating differential spousal survival rates (see Table 1).

There were clear educational disparities between urban and rural women. 70.7% of urban respondents had attained tertiary or postgraduate education, and only 47.8% of rural women achieved this level. Notably, 15.9% of rural women had no formal education, compared to just 1.7% in urban areas. These educational differences reflect longstanding rural–urban inequalities in access to schooling and adult literacy.

Age at menarche further underscored urban–rural health disparities. Urban women reported earlier menarche, with one-third (33.9%) beginning menstruation between ages 11 and 12, compared to only 11.2% of rural women. In contrast, over half (55%) of rural women experienced menarche at ages 15–16. While most respondents in both groups described their menstrual cycles as very regular before menopause, regularity was more commonly reported by rural women (93.8%) than urban women (84.5%).

In terms of reproductive history, rural women had higher fertility levels. None of the rural women reported being childless, whereas 7.7% of urban women had no children. Rural women were more likely to have had five or more children, with 61.0% having 5–6 children and 11.5% having up to 7–8 children. Breastfeeding was nearly universal across both settings, though rural women breastfed for longer durations: 96.4% reported breastfeeding for one year or more, compared to 77.4% of urban women. Family planning usage was similar in both settings, with approximately one-fifth of women reporting ever using contraception. However, urban women preferred hormonal methods (50%), while rural women more often used non-hormonal (55.6%) or barrier methods (25.9%). Miscarriage history was more frequently reported by urban women (38.9%) than rural women (26.8%), a difference that may reflect variations in access to healthcare, reproductive monitoring, and awareness of pregnancy loss.

### Source: authors

The age at menopause was initially grouped into two-year intervals (e.g., 42–43, 44–45) to allow for a more precise description of the distribution of menopausal age within the sample. Narrow interval grouping is commonly used in demographic and epidemiological studies to capture subtle variations in age patterns before broader analytical categories are applied.

### Menopausal timing and age by residential area

**Fig. 1:**
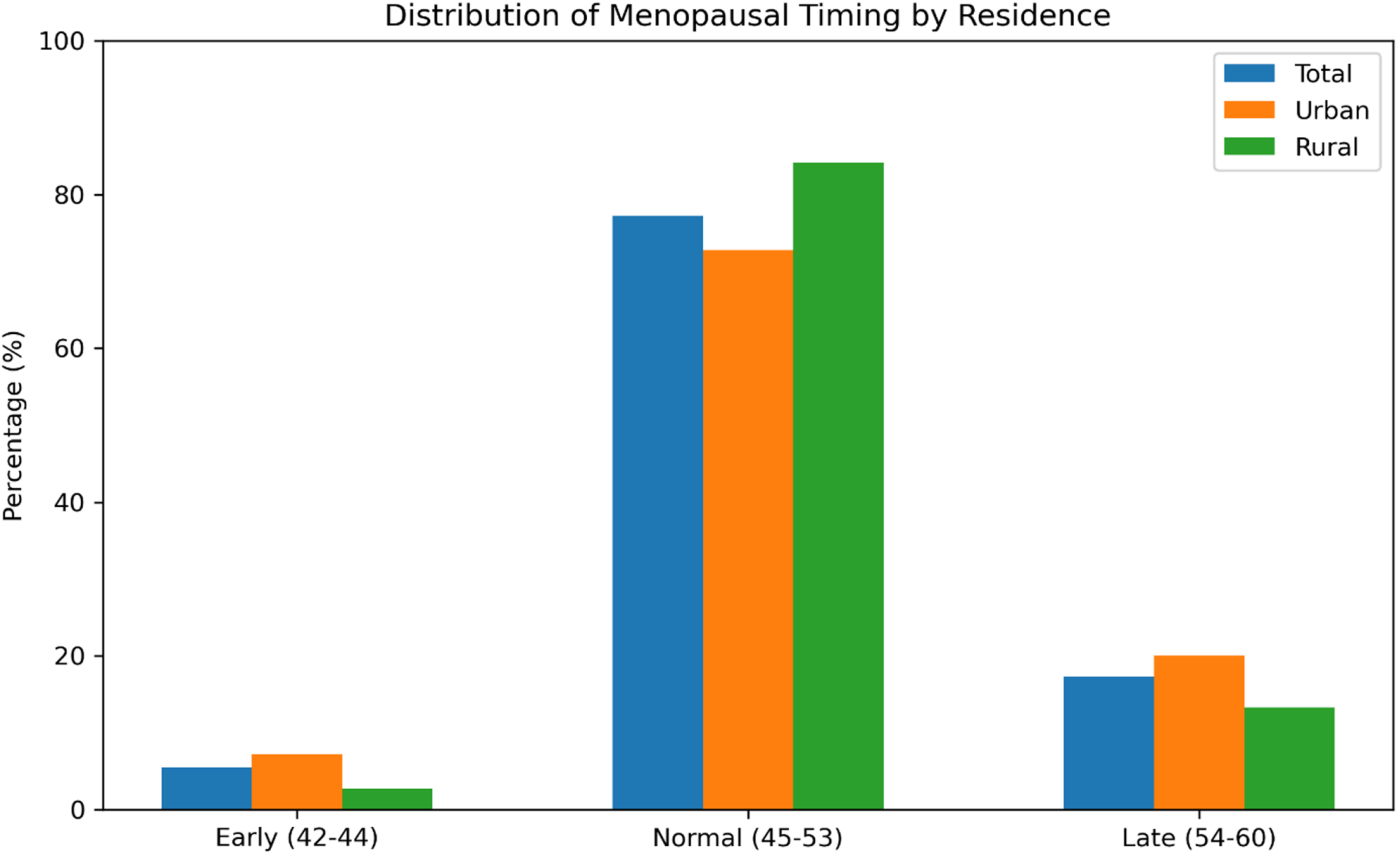
Timing of menopause

The timing of menopause among women in urban and rural areas in Nigeria was assessed using the average age at natural menopause. The overall mean age at menopause among the 294 respondents was 49.6 years (± 4.0) (Table 1). Based on the computed mean age at menopause and its standard deviation, menopause occurring between ages 45 and 53 years was classified as normal in this study. This range was derived from the statistical distribution of the data, where the mean age at menopause was approximately 49 years with a standard deviation of about 4 years,

Consequently, menopause occurring before age 45 was categorized as early menopause, while menopause occurring after age 53 was classified as late menopause (see Fig. 1). This classification allowed the study to identify variations in menopausal timing relative to the central tendency of the sample.

When disaggregated by place of residence, urban women experienced menopause slightly earlier, with a mean age of 49.3 years (± 3.9), compared to 49.9 years (± 4.3) among rural women. The majority of respondents (77.2%) experienced menopause within the normal age range of 45–53 years, while 5.5% experienced early menopause (42–44 years), and 17.3% experienced late menopause (54–60 years).

Residential differences in the distribution of menopausal timing were observed. Among urban women, 7.2% experienced early menopause and 20% experienced late menopause, compared with 2.7% and 13.3%, respectively, among rural women. Conversely, a higher proportion of rural women (84.1%) fell within the normal menopausal age range, compared to 72.8% of urban women.

### Association between reproductive history variables and menopausal timing among urban and rural women

Table 2 shows the association between reproductive history characteristics and menopausal timing examined among 181 urban and 113 rural women in Nigeria. We noted distinct patterns across residential settings, with significant relationships between menopausal timing and several socioeconomic variables. In urban areas, menopausal timing was statistically significantly associated (*p* < 0.05) with multiple variables, including age at menarche, menstrual cycle regularity, number of pregnancies and children, breastfeeding practices, family planning use, and history of miscarriage.

**Table 2:** Association between socioeconomic variables and menopausal timing among urban and rural women

Spatial Unit Menopausal Timing	Urban (%)				Rural (%)			
	*N* = 181				*N* = 113			
	Early	Normal	Late	χ²	Early	Normal	Late	χ²
Variables								
Age at Menarche				115.7**				47.9**
* 11–12*	0(0.0)	48(37.3)	12(34.3)		0(0.0)	3(3.8)	7(77.8)	
* 13–14*	1(7.7)	44(34.1)	18(51.4)		0(0.0)	26(33.3)	2(22.2)	
15–16	5(38.5)	32(24.8)	5(14.3)		2(100)	47(60.2)	0(0.0)	
* 17–18*	0(0.0)	5(3.9)	0(0.0)		0(0.0)	2(2.6)	0(0.0)	
* 19–20*	7(53.8)	0(0.0)	0(0.0)		0(0.0)	0(0.0)	0(0.0)	
Regularity of the Menstrual cycle				21.2**				**4.8**
* Very regular*	6(46.2)	111(84.1)	36(100)		2(66.7)	89(93.7)	15(100)	
* Irregular*	7(53.8)	21(15.9)	0(0.0)		1(33.3)	6(6.3)	0(0.0)	
Number of pregnancies				74.1**				62.8**
* 0*	0(0.0)	7(5.6)	7(19.4)		0(0.0)	0(0.0)	0(0.0)	
* 1–2*	0(0.0)	0(0.0)	1(2.8)		0(0.0)	0(0.0)	6(40.0)	
* 3–4*	1(7.7)	40(34.1)	0(0.0)		0(0.0)	15(15.9)	0(0.0)	
5–6	0(0.0)	35(27.8)	17(47.2)		0(0.0)	52(54.9)	1(6.7)	
* 7–8*	12(92.3)	36(28.5)	11(30.6)		2(66.7)	22(23.4)	8(53.4)	
* 9–10*	0(0.0)	8(6.4)	0(0.0)		1(33.3)	5(5.3)	0(0.0)	
Number of Children				74.4**				66.1**
* 0*	0(0.0)	7(5.3)	7(19.4)		0(0.0)	0(0.0)	0(0.0)	
* 1–2*	0(0.0)	19(14.4)	1(2.8)		0(0.0)	2(2.1)	6(40.0)	
3–4	8(61.5)	53(40.2)	0(0.0)		0(0.0)	23(24.2)	0(0.0)	
* 5–6*	5(38.5)	52(39.4)	28(77.7)		3(100)	64(67.4)	2(13.4)	
* 7–8*	0(0.0)	1(0.8)	0(0.0)		0(0.0)	6(6.3)	7(46.7)	
Breastfeeding				9.8**				**0.2**
* No*	0(0.0)	6(4.8)	7(19.4)		0(0.0)	1(1.1)	0(0.0)	
* Yes*,* all my children*	13(100)	119(95.2)	29(80.6)		3(100)	94(98.9)	15(100)	
Period of Breastfeeding				39.5**				**0.9**
* None*	0(0.0)	0(0.0)	7(19.4)		0(0.0)	0(0.0)	0(0.0)	
* 6 months or less*	0(0.0)	6(5.0)	0(0.0)		0(0.0)	1(1.1)	0(0.0)	
* 6–11 months*	0(0.0)	25(21.0)	0(0.0)		0(0.0)	3(3.2)	0(0.0)	
* One year and above*	13(100)	88(73.9)	29(80.6)		3(100)	90(95.7)	15(100)	
Family Planning				18.9**				6.2**
* No*	13(100)	88(69.8)	36(100)		3(100)	69(73.4)	15(100)	
* Yes*	0(0.0)	38(30.2)	0(0.0)		0(0.0)	25(26.6)	0(0.0)	
Methods of Family planning used				**cons**				**cons**
* Natural*		12(24.0)				2(7.4)		
* Hormonal Method*		25(50)				3(11.1)		
* Barrier Method*		0(0.0)				7(25.9)		
* Non-Hormonal Method*		13(26.0)				15(55.6)		
History of Miscarriage				23.1**				11.3**
* No*	1(7.7)	76(60.3)	30(83.3)		0(0.0)	68(72.3)	14(93.3)	
* Yes*	12(92.3)	50(39.7)	6(16.7)		3(100)	26(27.7)	1(6.7)	

χ² =Chi-square Values; ** Significant value *p*<0.05

Urban women who experienced early menarche (ages 11–12) were more likely to enter menopause at a normal or late age, while those who began menstruating later (ages 15–16 or 19–20) disproportionately experienced early menopause. Regular menstrual cycles were strongly associated with later menopausal onset, whereas irregular cycles were more prevalent among those who experienced early menopause.

Reproductive history based on parity was also closely tied to menopausal timing. Urban women with a higher number of pregnancies (i.e., 7–8 pregnancies) and children (i.e., 3–4) were more likely to experience early menopause, whereas those with no pregnancies or children were disproportionately represented among late menopausal women. Conversely, a larger proportion of women with 5–6 children experienced menopause at a later age.

Breastfeeding patterns were similarly indicative: not breastfeeding or having short breastfeeding durations (under one year) was linked to late menopause, while breastfeeding all children, especially for one year or more, was associated with earlier menopausal onset. The use of family planning methods was significantly related to timing; urban women who used contraception, particularly hormonal methods, tended to experience menopause within the normal age range, whereas none of the women with early or late menopause reported contraceptive use.

Urban women with a history of miscarriage were significantly more likely to experience early menopause, while those without such a history were overrepresented among normal and late menopausal groups.

Among rural women, on the other hand, different but equally significant patterns emerged. Late menopause was most prevalent among women with no or primary education, larger family sizes, and those working as farmers or unemployed, while early and normal menopause were more common among women with tertiary or postgraduate education, moderate stress levels, and stable employment.

Like their urban counterparts, rural women who experienced later menarche (ages 15–16) overwhelmingly fell within the normal menopausal range. Those with earlier menarche (ages 13–14) were more likely to undergo late menopause. Very regular menstrual cycles were strongly associated with later menopause, mirroring the urban trend.

In terms of fertility history, early menopause was observed among women with higher numbers of pregnancies (7–8), whereas those with 3–4 pregnancies or children experienced menopause within the normal age range. Remarkably, rural women with no children or only 1–2 children were more likely to have late menopause, although this group was numerically small.

Unlike the urban context, breastfeeding patterns and duration did not significantly affect menopausal timing among rural women. Nearly all rural respondents breastfed their children, predominantly for one year or longer, which may explain the lack of statistical variation. However, use of family planning methods remained a significant variable; those who never used any contraceptive method were more likely to experience late menopause. The history of miscarriage also showed a strong association in rural areas. Women who had miscarried were more likely to experience early or normal menopause, while those without such history were more represented among late menopausal cases.

### Influences of reproductive history variables on menopausal timing

The multinomial logistic regression model demonstrated strong predictive accuracy, correctly classifying menopausal timing at 82.5% in urban areas and 94.3% rural areas. Table 3 presents the odds ratios (ORs) for early and late menopause relative to normal menopausal timing, which serves as the reference category, based on the multinomial logistic regression model.

**Table 3:** Reproductive history influences on menopausal timing among urban and rural women

Residential Area	Urban *N* = 181		Rural *N* = 113	
Menopausal Timing	Early OR (95% CI)	Late OR (95% CI)	Early OR (95% CI)	Late OR (95% CI)
Variables				
Age at menarche	8.75(3.55–21.6)**	0.82(0.57–1.19)	1.89(0.32-11.0)	0.14(0.03–0.62)**
Number of pregnancies	0.33(0.09–1.20)	0.69(0.50–0.96)**	1.86(0.40-8.00)	1.85(1.10–3.10)**
Number of children	49.3(10.5-232.1)**	1.91(1.30–2.80)**	2.28(0.50–10.5)	1.84(0.90–3.80)

Reference category is normal menopause; ** Significant value *p*<0.05, 95% confidence Interval

Among urban respondents, several reproductive variables displayed varying associations with menopausal timing, although not all reached statistical significance. Age at menarche emerged as a strong and statistically significant predictor of early menopause (OR = 8.75, *p* < 0.05), suggesting that urban women who experienced earlier menarche were substantially more likely to experience earlier menopausal onset. Conversely, age at menarche showed no significant association with late menopause (OR = 0.82, *p* < 0.05).

The number of pregnancies demonstrated a significant, though inverse, association with late menopause (OR = 0.69, *p* < 0.05), indicating that women with more pregnancies had slightly reduced odds of late menopausal onset. The variable was not significant in predicting early menopause.

Most notably, the number of children was strongly and significantly associated with early menopause (OR = 49.3, *p* < 0.05), as well as late menopause (OR = 1.91, *p* < 0.05). These findings suggest a complex and nonlinear relationship between parity and menopausal timing, where higher parity may be linked to both early and late menopause, potentially due to cumulative physiological or hormonal effects across reproductive life.

Among rural respondents, the associations were less pronounced, though still informative. Age at menarche showed a significant negative association with late menopause (OR = 0.14, *p* < 0.05), implying that later menarche was associated with earlier menopausal transition in rural settings, opposite to the urban trend. The same variable showed no statistically significant effect on early menopause (OR = 1.89).

The number of pregnancies was a significant positive predictor of late menopause (OR = 1.85, *p* < 0.05), consistent with biological expectations that a longer reproductive span characterized by multiple pregnancies may delay menopausal onset. However, the association with early menopause was not significant.

Although the number of children was not statistically significant in the rural model, it still presented a moderate influence. Higher parity was associated with increased odds of both early (OR = 2.28, *p* < 0.05) and late (OR = 1.84, *p* < 0.05) menopause, echoing the mixed pattern seen among urban women, though not at a significant level.

## Discussion

This study investigated the relationship between reproductive history and menopausal timing among women in urban and rural settings, revealing both significant associations and spatial disparities. The results affirm that reproductive factors such as age at menarche, number of pregnancies, and parity (number of pregnancies and children) contribute to variations in menopausal timing, though these associations differ in strength and direction across residential areas.

The overall mean age at menopause in this study was 49.6 years, consistent with prior findings from sub-Saharan Africa [30]. However, urban women experienced menopause slightly earlier (mean: 49.3 years) than their rural counterparts (mean: 49.9 years), reflecting possible differences in reproductive behavior, lifestyle, nutrition, and access to health services. These findings are in line with global trends showing that urban living is often associated with earlier reproductive transitions due to stress, lower fertility, and increased exposure to modern healthcare [7, 31]. These patterns suggest that although the majority of women in both settings experienced menopause within the expected age range, urban women exhibited greater variability in menopausal timing. This could be influenced by lifestyle, environmental, or socioeconomic factors that differentiate urban living from the rural context. The findings indicate that there are important urban–rural differences in the demographic and reproductive histories of menopausal women. Urban women were more educated, had lower fertility, experienced menarche earlier, and used modern contraceptive methods, whereas rural women had higher fertility, later menarche, longer breastfeeding durations, and higher rates of widowhood. These factors are likely shaped by broader social, economic, and health system disparities across urban and rural environments.

These patterns emphasize the role of socioeconomic and reproductive life-course factors in shaping menopausal experiences and point to the need for context-specific interventions in women’s midlife health. In terms of reproductive history, urban women tended to have earlier menarche, fewer children, shorter breastfeeding durations, and higher contraceptive use patterns, likely influenced by education, employment, and delayed childbearing. These factors cumulatively appear to heighten the risk of early menopause, as shown in the multivariate logistic regression, where early menarche and high parity significantly increased the odds of early menopausal onset. This finding aligns with life-course and ovarian reserve theories, which posit that earlier reproductive maturation may accelerate the depletion of follicles, thus leading to earlier menopause [22].

In contrast, rural women displayed more uniformity in menopausal timing, with the majority experiencing menopause within the normal age range of 45–53 years. While reproductive exposures were less influential overall, late menopause in rural areas was significantly associated with the number of pregnancies and age at menarche. Notably, lower educational attainment, limited employment, and higher fertility characterized rural women, with a substantial proportion not using any form of family planning. These patterns echo findings from similar contexts [9] and suggest that social structure, access to reproductive healthcare, and cultural norms play a significant role in shaping the menopausal transition in rural environments.

Our findings also support previous evidence that socioeconomic and demographic factors interact with reproductive history to shape menopausal timing similar to Sharami et al. [16] who noted a relationship between reproductive history and menopausal symptoms. Urban women, despite having greater access to healthcare and education, showed more variability in menopausal timing, possibly due to more diverse reproductive life courses, lifestyle stressors, and occupational demands. On the other hand, the relative uniformity in rural menopausal patterns may reflect more traditional life trajectories and consistent reproductive exposure, even if shaped by structural inequalities.

These findings reinforce the importance of early-life reproductive events and cumulative fertility exposure as critical determinants of menopause timing. They also underscore the context-specific dynamics between reproductive life course and menopausal outcomes, shaped by urban-rural differences in lifestyle, access to care, and reproductive health behaviors.

Importantly, these patterns have policy and health implications. Early menopause is associated with increased risks of cardiovascular disease, osteoporosis, and overall morbidity, while late menopause has been linked to a higher risk of breast and endometrial cancers [32]. Understanding the predictors of menopausal timing across diverse settings can inform targeted public health interventions, especially in contexts with limited access to midlife healthcare services. The study also highlights how reproductive histories intersect with spatial inequalities to shape menopausal health in a specific low-and-middle income context. The findings support calls for integrated reproductive and midlife health services that are context-sensitive, particularly for underserved rural populations.

### Limitations of the study

This study employed non-probabilistic sampling to recruit participants, which inherently limits the representativeness of the sample. As a result, the findings may not be generalizable to all women. Specifically, the associations observed between reproductive factors and menopausal timing reflect patterns within the sampled participants and may be influenced by the characteristics of women who were accessible and willing to participate. This sampling approach may introduce selection bias, potentially over- or underestimating certain associations. Therefore, the results was more descriptive to provide insights into trends within the study population.

## Conclusions

This study reveals a complex interplay between reproductive experiences and menopausal timing, with marked urban-rural variations. Urban women were more educated, had lower fertility, experienced menarche earlier, and used more modern contraceptive methods, whereas rural women had higher fertility, later menarche, longer breastfeeding durations, and higher rates of widowhood. These factors are likely influenced by broader social, economic, and health system disparities across urban and rural environments. Furthermore, early menarche and high parity significantly increase the risk of early menopause in urban women, while late menopause in rural areas is modestly influenced by reproductive exposure. These findings highlight the need for life course and menopause specific approaches in women’s health interventions and policy. Integrating reproductive history into menopausal care frameworks can enhance early identification of at-risk groups and improve aging health outcomes in Nigeria.

## Supplementary Information

Supplementary Material 1.

Supplementary Material 2.

## Data Availability

All data generated or analysed during this study are included in this published article [and its supplementary information files].
