# Supplementary material for: Association between reproductive history and menopausal timing: exploring rural-urban differences in a cross-sectional survey
Source: BMC Womens Health. 2026 Apr 6;26:259. doi: 10.1186/s12905-026-04441-y (PMC13217787; doi:10.1186/s12905-026-04441-y)
Supplement: Supplementary file 1 — Supplementary Material 1. [file 12905_2026_4441_MOESM1_ESM.docx]

**APPENDIX A**

**QUESTIONNAIRE**

Department of Geography and Environmental Sustainability,

University of Nigeria, Nsukka,

Enugu State.

Dear Madam,

Questionnaire on **Menopausal Transition: Analyzing Rural-Urban Differences and Socioeconomic Influences on the Timing of Menopause among Nigerian Women.** We are conducting a study to analyze the timing of menopause among women in rural and urban areas of Enugu State, Nigeria. The study is for research purposes only, and your responses will remain strictly confidential. Participation is voluntary, and you can withdraw at any time.

Please answer the questions as accurately as possible. Your participation is greatly appreciated.

Yours Faithfully,

Ossai, O.G

**INSTRUCTION**

Fill in the blank spaces with the correct response or tick (√) in the appropriate spaces for the right option(s) as applicable to you.

**SECTION A: SOCIO-ECONOMIC CHARACTERISTICS OF THE RESPONDENTS BEFORE OR AT THE TIME OF MENOPAUSE**

1. Residential area: (a) Rural [ ] (b) Urban [ ]
2. Name of Residential area: __________________
3. State of Origin: _____________________
4. Age at menopause: ______________________ 4b. Current Age ________________
5. Marital status at menopause: (a) Married [ ] (b) Single [ ] (c) Divorced[ ] (d) Widowed.
6. Highest Educational Qualification achieved: (a) None [ ] (b) Primary [ ]

(c) Secondary [ ] (d) Higher [ ] (e) Postgraduate [ ]

1. Position in the Household: (a) Head [ ] (b) Wife [ ]
2. Employment status: (a) Employed [ ] (b) Unemployed [ ] (c) Retired [ ]
3. Occupation at menopause: (a) Employed by Government [ ] (b) Employed in a private firm[ ] (c) Self-employed[ ] (d) Farmer [ ] (e) others (specify)……..
4. Household size at menopause: ____________
5. Monthly Income at menopause: (a) below ~~N~~70,000 [ ] (b) ~~N~~70,000 – ~~N~~200,000 [ ] (c) above ~~N~~200,000 [ ]

**SECTION B: REPRODUCTIVE HISTORY**

1. At what age did you start menstruating (menarche)? _________________
2. How regular were your menstrual cycles before menopause?

(a) Very Regular (28-30 days) [ ](b) Irregular [ ] (c) Frequently missed periods [ ]

1. How many pregnancies have you had in your lifetime? ___________
2. Number of Children ______________
3. Did you breastfeed your children?

(a)Yes, all children [ ] (b) Yes, some children [ ](c) No [ ]

1. Did you use any family planning methods (contraceptives)?

(a) Yes [ ] (b) No [ ]

1. If Yes, specify method(s): ____________________
2. Have you had any gynecological surgeries (e.g., hysterectomy, ovary removal)?

(a) Yes [ ] (b) No [ ]

26. Do you have a history of miscarriage(s) or abortion(s)? (a) Yes [ ] (b) No [ ]
